# The genome sequence of a drosophilid fruit fly, *Hirtodrosophila cameraria *(Haliday, 1833)

**DOI:** 10.12688/wellcomeopenres.19850.1

**Published:** 2023-08-22

**Authors:** Darren J. Obbard, Wellcome Sanger Institute Tree of Life programme, Wellcome Sanger Institute Scientific Operations: DNA Pipelines collective, Tree of Life Core Informatics collective, Darwin Tree of Life Consortium

**Affiliations:** 1 Institute of Ecology and Evolution, The University of Edinburgh, Edinburgh, Scotland, EH9 3FL, UK

**Keywords:** Hirtodrosophila cameraria, a drosophilid fruit fly, genome sequence, chromosomal, Diptera

## Abstract

We present a genome assembly from an individual female *Hirtodrosophila cameraria* (a drosophilid fruit fly; Arthropoda; Insecta; Diptera; Drosophilidae). The genome sequence is 214.5 megabases in span. Most of the assembly is scaffolded into 4 chromosomal pseudomolecules. The mitochondrial genome has also been assembled and is 15.94 kilobases in length.

## Species taxonomy

Eukaryota; Metazoa; Eumetazoa; Bilateria; Protostomia; Ecdysozoa; Panarthropoda; Arthropoda; Mandibulata; Pancrustacea; Hexapoda; Insecta; Dicondylia; Pterygota; Neoptera; Endopterygota; Diptera; Brachycera; Muscomorpha; Eremoneura; Cyclorrhapha; Schizophora; Acalyptratae; Ephydroidea; Drosophilidae; Drosophilinae; Drosophilini; *Hirtodrosophila*; *Hirtodrosophila cameraria* ( Haliday, 1833) (NCBI:txid1262473).

## Background

**Figure 1.**
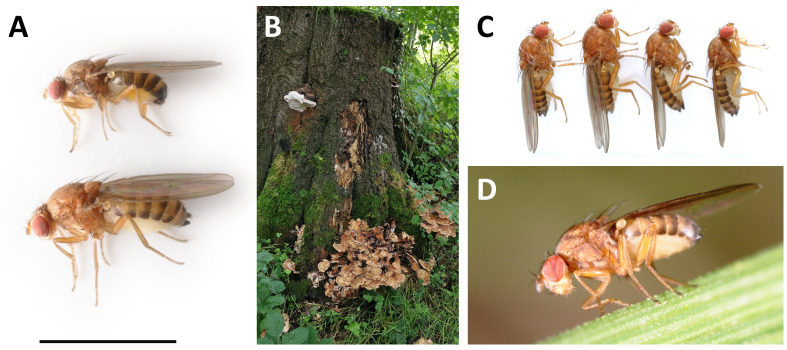
Photographs of *Hirtodrosophila cameraria* specimens and locale. **A**: Male (above) and female (below) *Hirtodrosophila cameraria* presented with a 3 mm scale bar. **B**: Tree stump from which the sequenced individuals were collected (Hermitage of Braid, Edinburgh, Scotland; 55.92, –3.20). **C**: The four unrelated wild-collected females provided to the Darwin Tree of Life project. Individual idHirCame1 (biospecimen SAMEA12110595) (left) was used for Hi-C sequencing, and individual idHirCame2 (biospecimen SAMEA12110596) (second left) was used for PacBio sequencing. **D**: Female *Hirtodrosophila cameraria* photographed above a toadstool in Perth & Kinross, Scotland.

Hirtodrosophila cameraria ( Haliday, 1833) is a medium sized (2.5–3.5 mm) pale greyish-brown drosophilid ‘fruit fly’ ( Figure 1A and B), distantly related to the laboratory model *Drosophila melanogaster.* It is one of three British and Irish species currently classified in the genus *Hirtodrosophila ( Chandler, 2023).* Originally placed in the genus *Drosophila* by Haliday, it was moved to the newly-elevated (sub-)genus *Hirtodrosophila* ( Grimaldi, 1990) by Bächli *et al.* (2004). However, relationships between *Drosophila* and *Hirtodrosophila* remain unclear, with the genera being paraphyletic with respect to each other and *Zygothrica* and *Mycodrosophila*, and with no single diagnostic morphological character available to separate them ( Bächli *et al.*, 2004; Finet *et al.*, 2021; Gautério *et al.*, 2020; Grimaldi, 1990). Nevertheless, *H. cameraria* itself is easy to identify among other similar British and Irish drosophilids, having plumose aristae with a single ventral branch behind the terminal fork and lacking a pre-apical seta on the mesotibia ( Bächli *et al.*, 2004).

Like its close relatives, *H. cameraria* is a specialist fungus breeder and in the UK the adults are easily collected or reared from toadstools and bracket fungi, including *Phallus impudicus*, *Lactarius quietus*, *Amanita rubescens*, *Russula cyanorantha* and *Paxillus* ( Charlesworth & Shorrocks, 1980; Gautério *et al.*, 2020; Shorrocks & Charlesworth, 1980). Adults have also been reported in association with the violet helleborine orchid *Epipactis purpurata* ( Roper, 2013), perhaps as a result of pheromones released by the plant to attract pollinators ( Policha *et al.*, 2019). *Hirtodrosophila cameraria* is broadly distributed in wooded areas across Europe, from the extreme north of Sweden, to Turkey in the east, and the Canary islands in the south west ( Bächli, 2023). In the UK, breeding is likely to be focused of toadstool flushes in the late summer and early autumn ( Charlesworth & Shorrocks, 1980), but adults can be caught at any time of year ( Basden, 1954), and are quite commonly recorded in the months of May to November ( GBIF Secretariat, 2022). It is not thought to be threatened and is in fact likely under-collected compared to its human-commensal relatives, as it rarely comes to fruit bait ( Basden, 1954).

Here we present a chromosomally complete genome sequence for *Hirtodrosophila cameraria*, derived from the DNA of two female specimens that were collected from a bracket fungus in the Hermitage of Braid, Edinburgh, as part of the Darwin Tree of Life Project. This genome sequence will help to resolve relationships among the Drosophilidae and will further build on the value of this family as a model clade for comparative genomics and molecular evolution. This project is a collaborative effort to sequence all named eukaryotic species in the Atlantic Archipelago of Britain and Ireland.

## Genome sequence report

The genome was sequenced from one female *Hirtodrosophila cameraria* ( Figure 1C) collected from Hermitage of Braid, Edinburgh, Scotland (55.92, –3.20). A total of 45-fold coverage in Pacific Biosciences single-molecule HiFi long reads was generated. Primary assembly contigs were scaffolded with chromosome conformation Hi-C data. Manual assembly curation corrected 78 missing joins or misjoins and removed 5 haplotypic duplications, reducing the assembly length by 0.32% and the scaffold number by 52.38%, and increasing the scaffold N50 by 101.28%.

**Figure 2:**
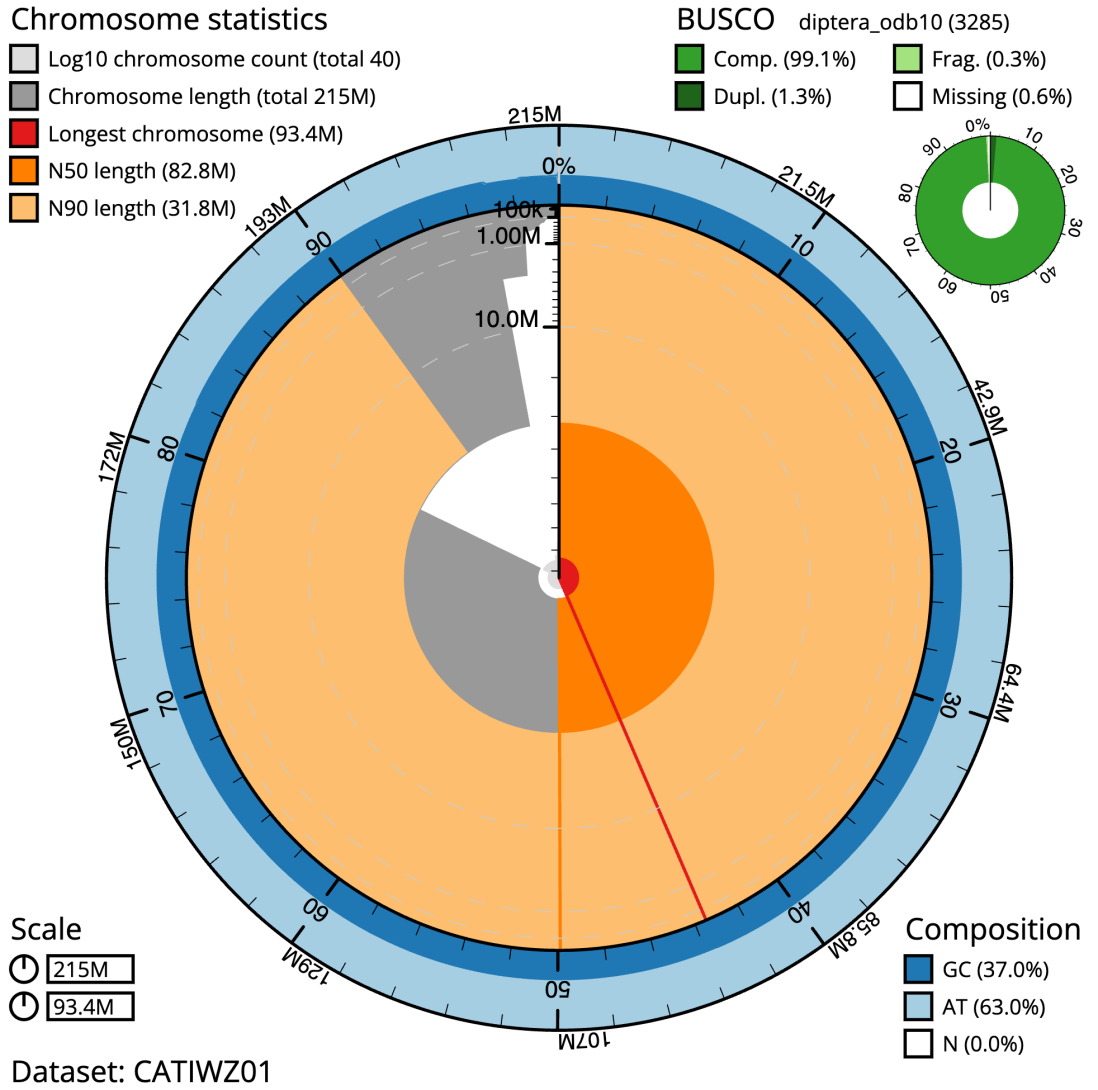
Genome assembly of *Hirtodrosophila cameraria*, idHirCame2.1: metrics. The BlobToolKit Snailplot shows N50 metrics and BUSCO gene completeness. The main plot is divided into 1,000 size-ordered bins around the circumference with each bin representing 0.1% of the 214,511,801 bp assembly. The distribution of scaffold lengths is shown in dark grey with the plot radius scaled to the longest scaffold present in the assembly (93,358,477 bp, shown in red). Orange and pale-orange arcs show the N50 and N90 scaffold lengths (82,766,246 and 31,806,066 bp), respectively. The pale grey spiral shows the cumulative scaffold count on a log scale with white scale lines showing successive orders of magnitude. The blue and pale-blue area around the outside of the plot shows the distribution of GC, AT and N percentages in the same bins as the inner plot. A summary of complete, fragmented, duplicated and missing BUSCO genes in the diptera_odb10 set is shown in the top right. An interactive version of this figure is available at https://blobtoolkit.genomehubs.org/view/idHirCame2.1/dataset/CATIWZ01/snail.

The final assembly has a total length of 214.5 Mb in 39 sequence scaffolds with a scaffold N50 of 82.8 Mb ( Table 1). Most (98.38%) of the assembly sequence was assigned to 4 chromosomal-level scaffolds. Chromosome-scale scaffolds confirmed by the Hi-C data are named in order of size ( Figure 2– Figure 5; Table 2). We did not identify the sex chromosome(s) as sequence data from the heterogametic sex was not available and homology is unreliable for sex chromosome identification in Diptera due to frequent sex chromosome turnover ( Vicoso & Bachtrog, 2015). While not fully phased, the assembly deposited is of one haplotype. Contigs corresponding to the second haplotype have also been deposited. The mitochondrial genome was also assembled and can be found as a contig within the multifasta file of the genome submission.

**Table 1.** Genome data for *Hirtodrosophila cameraria*, idHirCame2.1.

Project accession data		
Assembly identifier	idHirCame2.1	
Species	*Hirtodrosophila cameraria*	
Specimen	idHirCame2	
NCBI taxonomy ID	1262473	
BioProject	PRJEB56615	
BioSample ID	SAMEA12110596	
Isolate information	idHirCame2, female (DNA sequencing) idHirCame1 (Hi-C scaffolding)	
Assembly metrics *		*Benchmark*
Consensus quality (QV)	57.9	*≥ 50*
*k*-mer completeness	99.99%	*≥ 95%*
BUSCO **	C:99.1%[S:97.8%,D:1.3%], F:0.3%,M:0.6%,n:3,285	*C ≥ 95%*
Percentage of assembly mapped to chromosomes	98.38%	*≥ 95%*
Sex chromosomes	Not assigned	*localised homologous pairs*
Organelles	Mitochondrial genome assembled	*complete single alleles*
Raw data accessions		
PacificBiosciences SEQUEL II	ERR10368987	
Hi-C Illumina	ERR10378038	
Genome assembly		
Assembly accession	GCA_949708635.1	
*Accession of alternate haplotype*	GCA_949708645.1	
Span (Mb)	214.5	
Number of contigs	419	
Contig N50 length (Mb)	1.0	
Number of scaffolds	39	
Scaffold N50 length (Mb)	82.8	
Longest scaffold (Mb)	93.4	

* Assembly metric benchmarks are adapted from column VGP-2020 of “Table 1: Proposed standards and metrics for defining genome assembly quality” from ( Rhie *et al.*, 2021). ** BUSCO scores based on the diptera_odb10 BUSCO set using v5.3.2. C = complete [S = single copy, D = duplicated], F = fragmented, M = missing, n = number of orthologues in comparison. A full set of BUSCO scores is available at https://blobtoolkit.genomehubs.org/view/idHirCame2.1/dataset/CATIWZ01/busco.

**Figure 3.**
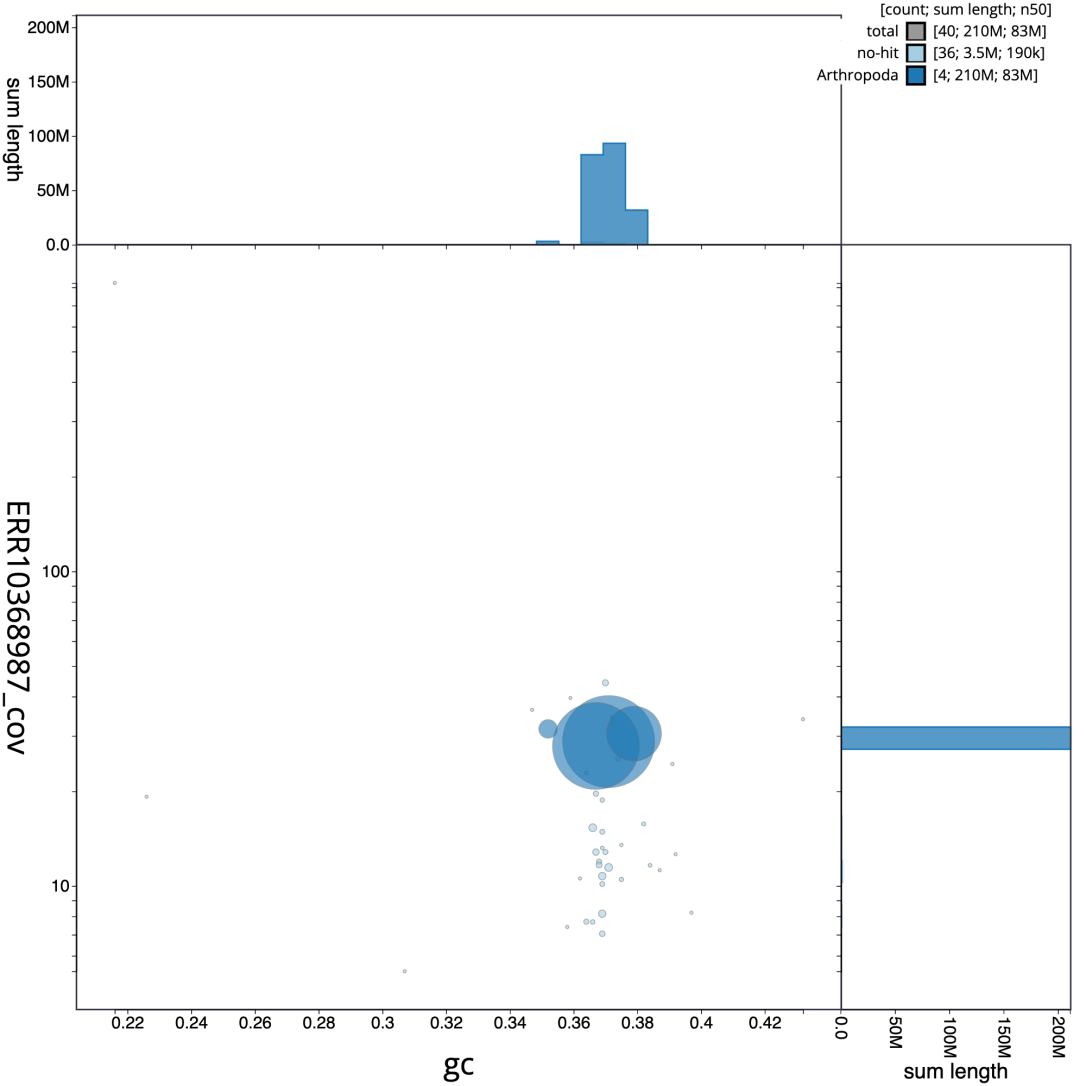
Genome assembly of *Hirtodrosophila cameraria*, idHirCame2.1: BlobToolKit GC-coverage plot. Scaffolds are coloured by phylum. Circles are sized in proportion to scaffold length. Histograms show the distribution of scaffold length sum along each axis. An interactive version of this figure is available at https://blobtoolkit.genomehubs.org/view/idHirCame2.1/dataset/CATIWZ01/blob.

**Figure 4.**
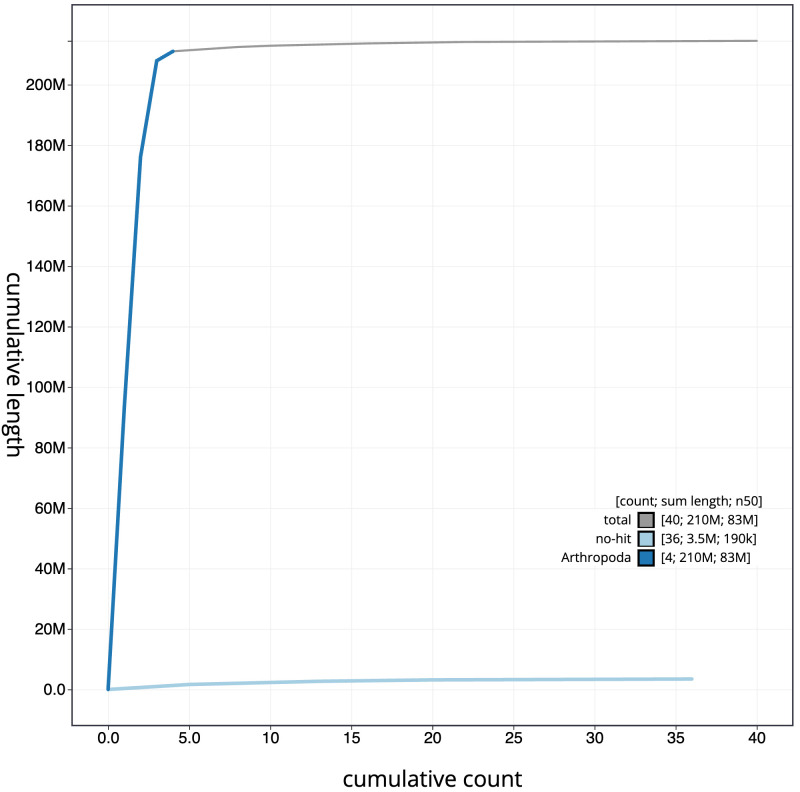
Genome assembly of *Hirtodrosophila cameraria*, idHirCame2.1: BlobToolKit cumulative sequence plot. The grey line shows cumulative length for all scaffolds. Coloured lines show cumulative lengths of scaffolds assigned to each phylum using the buscogenes taxrule. An interactive version of this figure is available at https://blobtoolkit.genomehubs.org/view/idHirCame2.1/dataset/CATIWZ01/cumulative.

**Figure 5.**
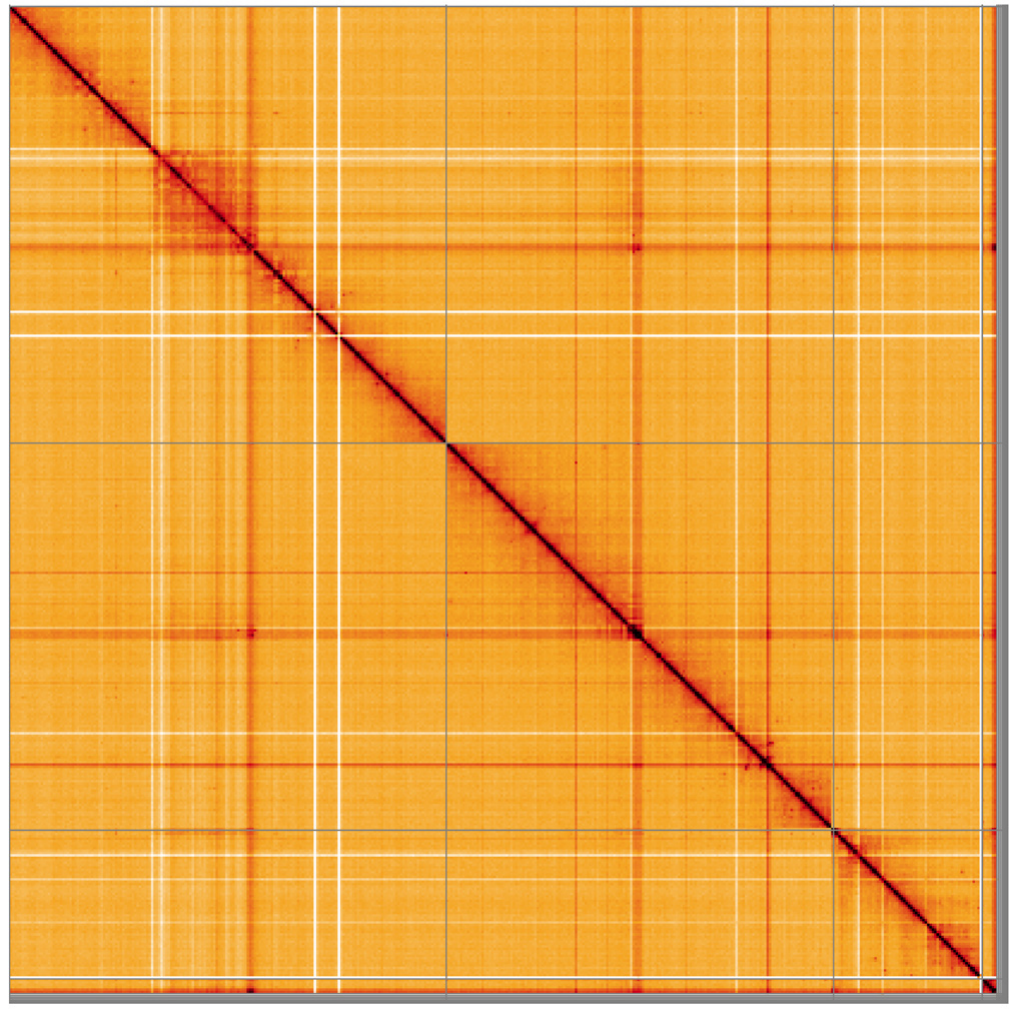
Genome assembly of *Hirtodrosophila cameraria*, idHirCame2.1: Hi-C contact map of the idHirCame2.1 assembly, visualised using HiGlass. Chromosomes are shown in order of size from left to right and top to bottom. An interactive version of this figure may be viewed at https://genome-note-higlass.tol.sanger.ac.uk/l/?d=WPeKseKJS1uSeK0NS7ZsOA.

**Table 2.** Chromosomal pseudomolecules in the genome assembly of *Hirtodrosophila cameraria*, idHirCame2.

INSDC accession	Chromosome	Length (Mb)	GC%
OX453089.1	1	93.36	37.0
OX453090.1	2	82.77	36.5
OX453091.1	3	31.81	38.0
OX453092.1	4	3.12	35.0
OX453093.1	MT	0.02	21.5

The estimated Quality Value (QV) of the final assembly is 57.9 with *k*-mer completeness of 99.99%, and the assembly has a BUSCO v5.3.2 completeness of 99.1% (single = 97.8%, duplicated = 1.3%), using the diptera_odb10 reference set ( *n* = 3,285).

Metadata for specimens, spectral estimates, sequencing runs, contaminants and pre-curation assembly statistics can be found at https://links.tol.sanger.ac.uk/species/1262473.

## Methods

### Sample acquisition and nucleic acid extraction

The specimen used for genome sequencing was a female *Hirtodrosophila cameraria* (biospecimen ID SAMEA12110596, individual idHirCame2; Figure 1C). The specimen used for Hi-C scaffolding was a female *Hirtodrosophila cameraria* (biospecimen ID SAMEA12110595, individual idHirCame1; Figure 1C). Both specimens were collected from Hermitage of Braid, Edinburgh, Scotland, UK (latitude 55.92, longitude –3.20) on 2021-10-04. The specimens were aspirated from a bracket fungus on a deciduous tree stump in an urban woodland. The anaesthetised flies were placed directly into collection tube, and frozen from live at –80°C. The specimens were collected and identified by Darren Obbard (University of Edinburgh).

DNA was extracted at the Tree of Life laboratory, Wellcome Sanger Institute (WSI). The idHirCame2 sample was weighed and dissected on dry ice. Tissue from the whole organism was disrupted using a Nippi Powermasher fitted with a BioMasher pestle. High molecular weight (HMW) DNA was extracted using the Qiagen MagAttract HMW DNA extraction kit. HMW DNA was sheared into an average fragment size of 12–20 kb in a Megaruptor 3 system with speed setting 30. Sheared DNA was purified by solid-phase reversible immobilisation using AMPure PB beads with a 1.8X ratio of beads to sample to remove the shorter fragments and concentrate the DNA sample. The concentration of the sheared and purified DNA was assessed using a Nanodrop spectrophotometer and Qubit Fluorometer and Qubit dsDNA High Sensitivity Assay kit. Fragment size distribution was evaluated by running the sample on the FemtoPulse system.

### Sequencing

Pacific Biosciences HiFi circular consensus DNA sequencing libraries were constructed according to the manufacturers’ instructions. DNA sequencing was performed by the Scientific Operations core at the WSI on a Pacific Biosciences SEQUEL II (HiFi) instrument. Hi-C data were also generated from whole organism tissue of idHirCame1 using the Arima2 kit and sequenced on the Illumina NovaSeq 6000 instrument.

### Genome assembly, curation and evaluation

Assembly was carried out with Hifiasm ( Cheng *et al.*, 2021) and haplotypic duplication was identified and removed with purge_dups ( Guan *et al.*, 2020). The assembly was then scaffolded with Hi-C data ( Rao *et al.*, 2014) using YaHS ( Zhou *et al.*, 2023). The assembly was checked for contamination and corrected as described previously ( Howe *et al.*, 2021). Manual curation was performed using HiGlass ( Kerpedjiev *et al.*, 2018) and Pretext ( Harry, 2022). The mitochondrial genome was assembled using MitoHiFi ( Uliano-Silva *et al.*, 2023), which runs MitoFinder ( Allio *et al.*, 2020) or MITOS ( Bernt *et al.*, 2013) and uses these annotations to select the final mitochondrial contig and to ensure the general quality of the sequence.

A Hi-C map for the final assembly was produced using bwa-mem2 ( Vasimuddin *et al.*, 2019) in the Cooler file format ( Abdennur & Mirny, 2020). To assess the assembly metrics, the *k*-mer completeness and QV consensus quality values were calculated in Merqury ( Rhie *et al.*, 2020). This work was done using Nextflow ( Di Tommaso *et al.*, 2017) DSL2 pipelines “sanger-tol/readmapping” ( Surana *et al.*, 2023a) and “sanger-tol/genomenote” ( Surana *et al.*, 2023b). The genome was analysed within the BlobToolKit environment ( Challis *et al.*, 2020) and BUSCO scores ( Manni *et al.*, 2021; Simão *et al.*, 2015) were calculated.

Table 3 contains a list of relevant software tool versions and sources.

**Table 3.** Software tools: versions and sources.

Software tool	Version	Source
BlobToolKit	4.1.7	https://github.com/blobtoolkit/blobtoolkit
BUSCO	5.3.2	https://gitlab.com/ezlab/busco
Hifiasm	0.16.1-r375	https://github.com/chhylp123/hifiasm
HiGlass	1.11.6	https://github.com/higlass/higlass
Merqury	MerquryFK	https://github.com/thegenemyers/MERQURY.FK
MitoHiFi	2	https://github.com/marcelauliano/MitoHiFi
PretextView	0.2	https://github.com/wtsi-hpag/PretextView
purge_dups	1.2.3	https://github.com/dfguan/purge_dups
sanger-tol/ genomenote	v1.0	https://github.com/sanger-tol/genomenote
sanger-tol/ readmapping	1.1.0	https://github.com/sanger-tol/readmapping/tree/1.1.0
YaHS	yahs- 1.1.91eebc2	https://github.com/c-zhou/yahs

### Wellcome Sanger Institute – Legal and Governance

The materials that have contributed to this genome note have been supplied by a Tree of Life collaborator. The Wellcome Sanger Institute employs a process whereby due diligence is carried out proportionate to the nature of the materials themselves, and the circumstances under which they have been/are to be collected and provided for use. The purpose of this is to address and mitigate any potential legal and/or ethical implications of receipt and use of the materials as part of the research project, and to ensure that in doing so we align with best practice wherever possible. The overarching areas of consideration are:

•   Ethical review of provenance and sourcing of the material

•   Legality of collection, transfer and use (national and international)

Each transfer of samples is undertaken according to a Research Collaboration Agreement or Material Transfer Agreement entered into by the Tree of Life collaborator, Genome Research Limited (operating as the Wellcome Sanger Institute) and in some circumstances other Tree of Life collaborators.

## Data Availability

European Nucleotide Archive: *Hirtodrosophila cameraria*. Accession number PRJEB56615; https://identifiers.org/ena.embl/PRJEB56615. ( Wellcome Sanger Institute, 2023) The genome sequence is released openly for reuse. The *Hirtodrosophila cameraria* genome sequencing initiative is part of the Darwin Tree of Life (DToL) project. All raw sequence data and the assembly have been deposited in INSDC databases. The genome will be annotated using available RNA-Seq data and presented through the Ensembl pipeline at the European Bioinformatics Institute. Raw data and assembly accession identifiers are reported in Table 1.

## Author information

Members of the Wellcome Sanger Institute Tree of Life programme are listed here: https://doi.org/10.5281/zenodo.4783585.

Members of Wellcome Sanger Institute Scientific Operations: DNA Pipelines collective are listed here: https://doi.org/10.5281/zenodo.4790455.

Members of the Tree of Life Core Informatics collective are listed here: https://doi.org/10.5281/zenodo.5013541.

Members of the Darwin Tree of Life Consortium are listed here: https://doi.org/10.5281/zenodo.4783558.
