# Supplementary material for: The genome sequence of a drosophilid fruit fly, Hirtodrosophila cameraria (Haliday, 1833)
Source: Wellcome Open Res. 2023 Aug 22;8:361. [Version 1] doi: 10.12688/wellcomeopenres.19850.1 (PMC11167333; doi:10.12688/wellcomeopenres.19850.1)
Supplement: Peer review history

# Reviewer report: Reviewer response for version 1

**DOI:** 10.21956/wellcomeopenres.21983.r84367

**Published:** 2024-06-11

**Authors:** Ludvik M Gomulski

**Affiliations:** 1 University of Pavia, Pavia, Italy

The manuscript reports the sequencing of the genome and mitochondrial genome from a single British female specimen of a drosophilid fly, *Hirtodrosophila cameraria*. The methods and analyses used are well described and appropriate and the datasets are readily accessible. 

 1. *Hirtodrosophila cameraria* should always be in italics - this is not so in the keywords and in the first sentence of the Background section.

 2. It is incorrect to refer to substances released by orchids that attract pollinators as 'pheromones'  - use 'semiochemicals' or 'allelochemicals'.

Are sufficient details of methods and materials provided to allow replication by others?

Yes

Is the rationale for creating the dataset(s) clearly described?

Yes

Are the datasets clearly presented in a useable and accessible format?

Yes

Are the protocols appropriate and is the work technically sound?

Yes

Reviewer Expertise:

insect genetics and behaviour

I confirm that I have read this submission and believe that I have an appropriate level of expertise to confirm that it is of an acceptable scientific standard.

# Reviewer report: Reviewer response for version 1

**DOI:** 10.21956/wellcomeopenres.21983.r80894

**Published:** 2024-05-20

**Authors:** Markus Friedrich

**Affiliations:** 1 Wayne State University, Detroit, Michigan, USA

This manuscript reports the sequencing and chromosome-level assembly of the genome of the fruitfly species *Hirtodrosophila cameraria* in the context of the Darwin Tree of Life project.

 Experimental design and data analysis are comprehensively described.

 Only three minor editorial issues:

 1.

*Hirtodrosophila cameraria*

 Italicize in the first sentence of the Background section.

 2.

 “Originally placed in the genus Drosophila by Haliday…”

 Reference?

 3.

 Figure 1

 The legend states “D: Female *Hirtodrosophila cameraria* photographed above a toadstool...”

 I can’t identify a toadstool or toadstool structure in panel D.

Are sufficient details of methods and materials provided to allow replication by others?

Yes

Is the rationale for creating the dataset(s) clearly described?

Yes

Are the datasets clearly presented in a useable and accessible format?

Yes

Are the protocols appropriate and is the work technically sound?

Yes

Reviewer Expertise:

Comparative genomics

I confirm that I have read this submission and believe that I have an appropriate level of expertise to confirm that it is of an acceptable scientific standard.
